# Diseleno[3,2‐*b*:2′,3′‐*d*]selenophene‐Containing High‐Mobility Conjugated Polymer for Organic Field‐Effect Transistors

**DOI:** 10.1002/advs.201900245

**Published:** 2019-04-26

**Authors:** Soo‐Young Jang, In‐Bok Kim, Minji Kang, Zuping Fei, Eunhwan Jung, Thomas McCarthy‐Ward, Jessica Shaw, Dae‐Hee Lim, Yeon‐Ju Kim, Sanjay Mathur, Martin Heeney, Dong‐Yu Kim

**Affiliations:** ^1^ Research Institute for Solar and Sustainable Energies (RISE) Gwangju Institute of Science and Technology (GIST) 123 Cheomdangwagi‐ro, Buk‐gu Gwangju 61002 Republic of Korea; ^2^ Department of Chemistry and Centre for Plastic Electronics Imperial College London Exhibition Rd London SW7 2AZ UK; ^3^ Inorganic and Materials Chemistry University of Cologne Cologne 50939 Germany; ^4^ School of Materials Science and Engineering (SMSE) Gwangju Institute of Science and Technology (GIST) 123 Cheomdangwagi‐ro, Buk‐gu Gwangju 61002 Republic of Korea

**Keywords:** conjugated polymers, diseleno[3,2‐*b*:2′, 3′‐*d*]selenophenes, intermolecular interactions, organic field‐effect transistors

## Abstract

The synthesis of a diseleno[3,2‐*b*:2′,3′‐*d*]selenophene (DSS) composed of three fused selenophenes is reported and it is used as a building block for the preparation of a high hole mobility conjugated polymer (PDSSTV). The polymer demonstrates strong intermolecular interactions even in solution, despite steric repulsion between the large Se atom in DSS and adjacent (C_β_)–H atoms which leads to a partially twisted confirmation PDSSTV. Nevertheless, 2D grazing incidence X‐ray diffraction (2D‐GIXD) analysis reveals that the polymer tends to align in a highly ordered edge‐on orientation after thermal annealing. The polymer demonstrates promising performance in a field‐effect transistor device with saturated hole mobility up to 2 cm^2^ V^−1^ s^−1^ obtained under relatively low gate voltages of −30 V. The ultilization of a Se‐containing fused aromatic system, therefore, appears to be a promising avenue for the development of high‐performance conjugated polymers.

Organic field‐effect transistors (OFETs) based on conjugated polymers have been widely studied and significant progress has been reported in their device performance.^1^, ^2^, ^3^ A key factor underlying this progress has been the improvement of the field‐effect mobility of these conjugated polymers, with various structural strategies applied including the interdigitation of alkyl side chains to ensure high crystallinity,^4^ planarization of backbones to suppress angular torsions,^5^ and donor–acceptor (D–A) alternation to reinforce intermolecular interactions.^6^, ^7^ In addition, the incorporation of larger chalcogen atoms into conjugated backbones can also improve charge transport ability.^8^, ^9^ In particular the replacement of thiophene with selenophene has attracted significant interest. The larger and more polarizable orbitals of selenium (Se) can enhance efficient intermolecular interactions, thereby facilitating charge transport between polymer chains.^2^, ^10^, ^11^, ^12^, ^13^, ^14^, ^15^

Along with chalcogen introduction, the incorporation of a fused ring structure comprising several aromatic rings combined into one large molecule is another typical method of reinforcing intermolecular interactions.^16^, ^17^ The flat and rigid structure of the resulting polymers not only promotes molecular orbital overlap between polymer chains but also reduces rotational torsion within conjugated backbones.^18^

In this paper, we synthesized and characterized a high‐hole‐mobility conjugated polymer (PDSSTV) containing a newly developed diseleno[3,2‐*b*:2′,3′‐*d*]selenophene (DSS) unit, which comprises three fused selenophenes to afford a rigid and planar aromatic. Although derivatives of the corresponding sulfur (S) version (dithieno[3,2‐*b*:2′,3′‐*d*]thiophene) have been widely studied as components of conjugated systems for optoelectronic materials,^19^, ^20^, ^21^ the Se analogue DSS has, to the best of our knowledge, not been previously reported. Since DSS does not contain any solubilizing sidechains, we introduced an (*E*)‐1,2‐(3,3′‐dioctadecyl‐2,2′‐dithienyl)‐ethylene (TV) unit as a co‐monomer. Both monomers are electron‐rich aromatics, resulting in the formation of an all donor–donor‐type conjugated polymer. Such all donor copolymers have previously been shown to exhibit promising OFET performance without the undesirable deviations from ideal device behavior (e.g., ambipolar character) often observed for many D–A systems.^22^, ^23^ Here we show that PDSSTV displays strong aggregation behavior, even in solution, as a result of the strong intermolecular interactions. This occurs despite the fact that the conjugated backbone of PDSSTV is predicted to have a large dihedral angle (32.1°) between the two monomers, as a result of steric interactions between large Se atom and the adjacent thiophene. Thin films of the polymer were found to adopt a highly crystalline structure by 2D grazing incidence X‐ray diffraction (2D‐GIXD) analysis. In OFETs featuring a newly developed polystyrene/poly(vinylidene fluoride‐trifluoroethylene‐chlorofluoroethylene) (PS/P(VDF‐TrFE‐CFE)) bilayer dielectric system, PDSSTV demonstrated a maximum mobility of 2.1 cm^2^ V^−1^ s^−1^ at an operating voltage (−30 V) much lower than those required for other high‐mobility conjugated polymers (typically up to −100 V).^2^, ^24^ Moreover, mobilities measured in the saturation regime exhibited ideal gate voltage–independent behavior, which resulted in increased device reliability.

PDSSTV was synthesized by a microwave‐assisted Stille polycondensation (**Scheme** 1). The novel DSS was prepared from 5,5′‐bis(trimethylsilyl)‐3,3′‐dibromo‐2,2′‐biselenophene 1)^11^ by a Pd catalyzed Se insertion reaction using (Bu_3_Sn)_2_Se 2) to afford DSS‐TMS 3) in 48% yield. It was then desilylated using TBAF to afford DSS 4) in 71% yield, followed by bromination in the 2,6 position by treatment with NBS in a yield of 79%. The resulting monomer DSS‐Br 5) was polymerized with the co‐monomer 5,5′‐ditrimethylstannyl‐(*E*)‐1,2‐(3,3′‐dioctadecyl‐2,2′‐dithienyl)ethylene 6)^22^ in the presence of Pd_2_(dba_3_)/P(o‐tol)_3_ under microwave heating. After precipitation and solvent washing to remove impurities and catalyst residues, the resulting polymer was extracted into hot chloroform and precipitated to afford PDSSTV in good yield (79%).

**Scheme 1:**
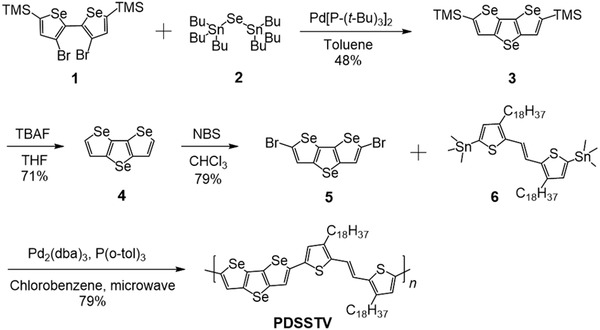
Synthesis of monomer 5 and PDSSTV.

The resulting polymer had a reasonable number average molecular weight of 19.1 kDa with a dispersity of 2 as measured by gel permeation chromatography in hot (150 °C) 1,2,4‐trichlorobenzene against polystyrene (Figure S4, Supporting Information). All the synthetic details including 5, 6, and PDSSTV are described in Experimental Section in Supporting Information. PDSSTV is highly soluble in chlorinated aromatic solvents including chlorobenzene and *o*‐dichlorobenzene when viewed with the bare eye. Differential scanning calorimetry revealed that only thermal transitions of the linear octadecyl alkyl chains in TV units were observed at ≈22 °C (*T*_c_) and 37 °C (*T*_m_) as exo/endothermic peaks, respectively. The absence of thermal transitions which can be originated from conjugated backbones was likely due to strong intermolecular interactions between the polymer chains.^22^ The thermal stability of PDSSTV was further assessed by thermogravimetric analysis, which showed that a moderate weight loss of 5% was observed at 356 °C (Figure S5, Supporting Information).

The optical properties of the thus obtained polymer in thin film (**Figure** 1a) and solution forms (Figure 1a,b) were probed by UV–vis absorption spectroscopy. Notably, the spectrum of the spin‐coated film displayed a main absorption at 617 nm, with a pronounced vibronic shoulder around 671 nm and the optical bandgap was estimated as 1.70 eV. Compared to the solution spectra, the thin‐film vibronic shoulder was increased in intensity, which is suggestive of increased order in the solid state. The intensity of the above peak increased slightly upon annealing at 250 °C, likely as a result of the thermally induced rearrangement of polymer chains.^25^ Moreover, the bathochromic shift of peaks occurred upon going from the solution phase to the film phase was negligible, which indicate that the presence of strong intermolecular interactions between polymer chains resulted in the pre‐aggregation of these chains in solution.^22^ However, the gradual blue‐shift observed in temperature‐dependent absorption spectra upon increasing the solution temperature from 40 to 150 °C in Figure 1b suggested that these polymer aggregates could be broken up by heating.^19^, ^22^, ^26^

**Figure 1:**
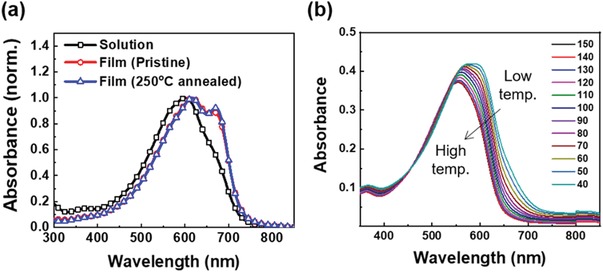
UV–vis absorption spectra of a) PDSSTV solution, pristine film, and film annealed at 250 °C and b) PDSSTV solutions with temperatures from 150 to 40 °C (≈4 × 10^−6^ m, chlorobenzene solution).

The energy levels of PDSSTV were calculated by cyclic voltammetry (CV) analysis using tetrabutylammonium hexafluorophosphate as an electrolyte (Figure S6, Supporting Information). Based on the onset of oxidation peak, the highest occupied molecular orbital (HOMO) energy level was calculated as −4.88 eV, which was close to that of P3HT (−4.83 eV) under identical conditions. The reduction onset was not clearly observable by CV, so the lowest unoccupied molecular orbital (LUMO) energy level was estimated to be −3.18 eV based on the optical bandgap and HOMO energy level.

Density functional theory (DFT) analysis was performed on a trimer of PDSSTV in order to understand the molecular geometry and energy levels (methyl groups were used as the alkyl side chains to save computational time). It is interesting to note that whereas the rigid and planar DSS and TV monomers resulted in both essentially co‐planar structure, the corresponding trimer was predicted to exhibit some torsional disorder in the backbone, as shown in **Figure** 2a. In the trimer, the presence of angular torsion (32.1°) between monomeric units (DSS and TV) was mainly ascribed to the steric repulsion between the large Se atom and the (C_β_)–H atom of the neighboring TV unit (highlighted by a red dotted oval in Figure 2b).^26^, ^27^, ^28^, ^29^ Moreover, in contrast to the negatively charged S of the TV unit, the Se of the DSS featured an almost neutral charge distribution (Figure 2c), which suggested that the attractive interaction between C_β_ and Se was minimal. However, despite the abovementioned distorted conformation, the absorption spectra of PDSSTV in solution and film forms were indicative of strong intermolecular interactions (Figure 1), which was attributed to the strong Se–Se attraction arising from the large overlap of Se orbitals.^8^, ^9^, ^10^, ^11^, ^12^, ^13^ In addition, charge neutrality within the DSS unit was believed to allow the nonbonding electrons of Se to be easily incorporated into the π‐conjugated system and thus promote charge delocalization. Figure S7 in the Supporting Information shows the well‐distributed HOMO and LUMO of the trimer, revealing that charge carriers could be sufficiently delocalized through the conjugated system.

**Figure 2:**
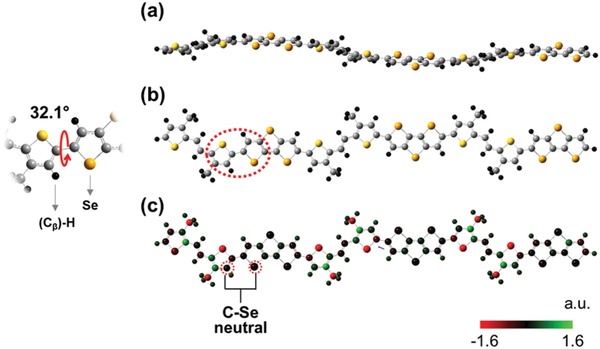
Structural simulation of the PDSSTV trimer. a) Side view, b) top view, and c) charge distribution (simulation model: B3LYP‐D3/6‐311++G*).

The crystallinity and molecular orientation of PDSSTV were investigated by 2D‐GIXD analysis (**Figure** 3). Figure 3a,b shows the 2D‐GIXD images and line cuts of pristine PDSSTV, revealing that the polymer has an ordered structure with an edge‐on orientation by showing the out‐of‐plane 1st to 4th order diffraction corresponding to a *d*‐spacing of 25.1 Å. The polymer annealed at 250 °C (in Figure 3c,d) exhibited a reduced *d*‐spacing of 24.2 Å with a significant increase in the intensity of the diffraction peak compared with the pristine state. Also, the intensity of the (010) in‐plane diffraction peak (*d* = 3.69 Å) increased after annealing, which means the thermal treatment reinforced the pi‐stacking of the polymer chains. In order to further confirm the effect of heat treatment on the relative crystallinity of the polymer thin films, the crystalline coherence length, which refers to the dimension of the polymer crystallites, was calculated. In both (100) lamella *d*‐spacing and (010) π–π stacking directions, the coherence length increased from 6.07 to 18.89 nm and from 7.67 to 9.23 nm respectively, demonstrating the increase in size of the ordered domains of the polymer upon thermal annealing.^30^ This annealing‐induced enhancement of the crystallinity in company with the intensity increase of the vibronic peak in the UV–vis absorption spectrum of the polymer film (Figure 1a) can be explained by the results of the thermally induced rearrangement of polymer backbones.^31^ Overall, annealed PDSSTV chains featured a highly ordered edge‐on orientation, which promoted horizontal charge transport in OFETs.^2^, ^22^

**Figure 3:**
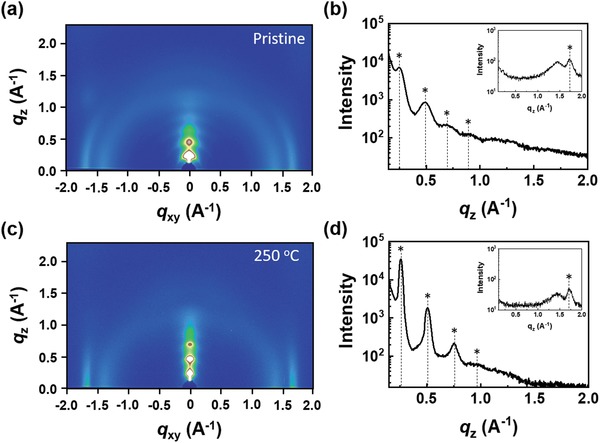
a,c) 2D‐GIXD images, b,d) out‐of‐plane diffraction profiles (with in‐plane profile inset) of pristine (a,b) and annealed (at 250 °C); c,d) PDSSTV films.

The charge carrier mobility of PDSSTV as a semiconducting channel material was probed by fabricating OFETs with a top‐gate/bottom‐contact (TG/BC) architecture, with a detailed description of the fabrication process given in the Experimental Section (S2). In particular, a polystyrene/vinylidene fluoride‐trifluoroethylene‐chlorofluoroethylene terpolymer (PS/P(VDF‐TrFE‐CFE)) dielectric bilayer was used as a newly developed polymer dielectric system. Both of these materials (PS and P(VDF‐TrFE‐CFE)) are solution‐processable and are well known to be low‐/high‐*k* dielectrics, respectively.^32^, ^33^ This combination of low‐ and high‐*k* dielectrics has been occasionally utilized in OFETs to reduce the energetic disorder of trap states at the surface of the active layer arising from the randomly oriented dipoles of high‐*k* dielectrics.^34^, ^35^, ^36^, ^37^ The TG/BC OFET structure and transfer/output curves for best‐performing devices (pristine and annealed at 250 °C) are illustrated in **Figure** 4, and the extracted numerical parameters are listed in **Table** 1. Upon annealing at 250 °C, the maximum saturation mobility increased from 0.27 to 2.1 cm^2^ V^−1^ s^−1^ because of the concomitant increase of the degree of molecular ordering, as confirmed by UV–vis absorption spectra and GIXD patterns. Importantly, whereas most previously reported high‐mobility semiconducting polymers required high operating biases of up to ‐100 V,^2^, ^24^ the use of a PS/P(VDF‐TrFE‐CFE) dielectric system allowed the use of lower biases, since low‐*k* PS prevented the formation of trap states that could result from the use of high‐*k* P(VDF‐TrFE‐CFE), while the latter dielectric itself allowed OFETs to be operated at a relatively low voltage (in this study, −30 V). Furthermore, the mobilities were almost independent of gate voltage (Figure S8, Supporting Information), although the reverse is commonly observed for high‐performance OFETs because of the presence of localized states in the bulk channel, defects at the channel/dielectric interface, or current leakage at the gate dielectric.^38^ Based on the abovementioned results, we suggest that charge transport in bulk PDSSTV film at low operating voltages is facilitated by the Se–Se interaction–induced strong attraction between polymer chains and high film crystallinity, further hypothesizing that the DSS unit could be used for broad applications in the development of high‐mobility conjugated molecules for OFETs. Meanwhile, the corresponding S version of the PDSSTV^22^ was fabricated into OFETs with the same device configuration (TG/BC, PS/P(VDF‐TrFE‐CFE) bi‐dielectric) to compare the effect of S and Se on the charge carrier mobility of each polymer (Figure S9 and Table S1, Supporting Information). The average saturated hole mobility of the S version polymer (250 °C thermal annealed) measured 2.03 cm^2^ V^−1^ s^−1^, similar with that of PDSSTV (average: 1.58 cm^2^ V^−1^ s^−1^, in Table 1). As mentioned in DFT analysis section, Se induces large angular torsion (32.1°) between the monomer units owing to its large size, which could hamper the π‐orbital overlap along the polymer chains despite the possibility of strong intermolecular interaction induced by its large and polarizable orbitals. The OFETs with different device configuration (BG/BC) were also checked, and they showed relatively lower mobility than that of TG/BC devices (Figure S10 and Table S2, Supporting Information). Therefore, there is a geometry dependence of the OFET device performance which can be attributed to the contact resistance or poor PDSSTV film formation on the hydrophobic HMDS dielectric, etc.^39^, ^40^ The surface morphology of the polymer film was probed by atomic force microscopy (AFM), and pristine and annealed (250 °C) films were shown to exhibit similar root mean square roughness values of 5.8 and 5.9 nm, respectively, in **Figure** 5. However, the size of aggregates decreased upon annealing, even though polymer crystallinity concomitantly increased (Figure 3). Considering the fact that the presence of large aggregates in the channel layer could lead to high contact resistance or device failure due to poor channel‐dielectric contact, the annealing of PDSSTV was concluded to favorably influence its molecular ordering and surface morphology.

**Figure 4:**
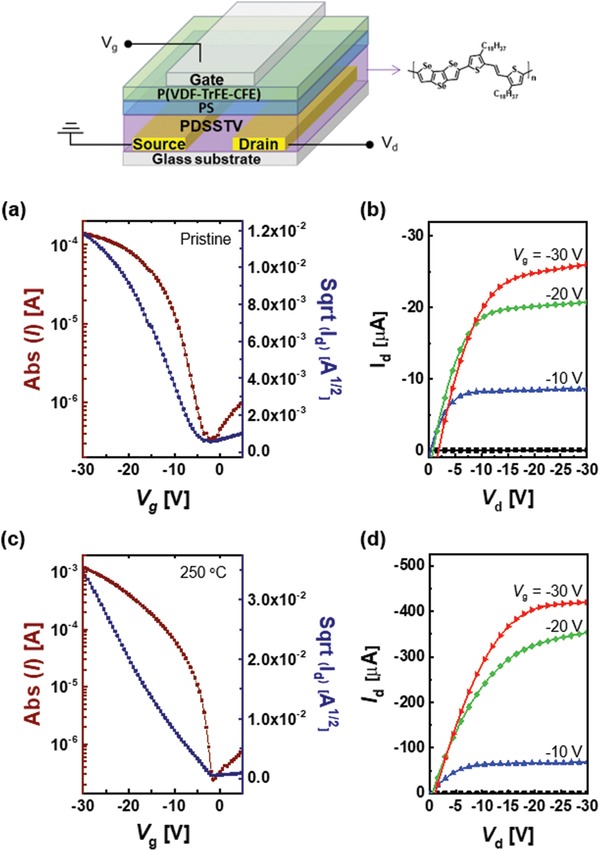
Top: Structure of PDSSTV‐based TG/BC OFETs. a,c) Transfer curves and b,d) output curves of devices comprising pristine (a,b) and annealed (250 °C); (c,d) PDSSTV films.

**Figure 5:**
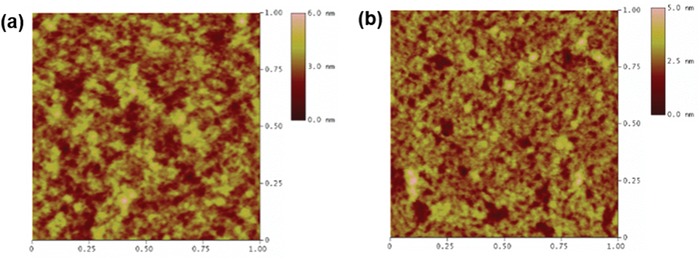
AFM images of pristine a) and 250 °C annealed b) PDSSTV films (height mode).

**Table 1:** Performance of PDSSTV‐based OFETs

Annealing temp. [°C]	Mobility [cm^2^ V^−1^ s^−1^]		Threshold voltage [V]	On/Off ratio
	Average/ standard deviation	Maximum		
Pristine	0.25 / 0.027	0.27	−4.5	>10^3^
250	1.58 / 0.27	2.11	−7.2	>10^3^

In summary, we report the synthesis of a novel 3‐ring fused selenophene aromatic. We have successfully prepared a high‐mobility conjugated polymer (PDSSTV) using DSS and TV as monomeric units. Although DFT analysis demonstrated that the steric repulsion between Se and adjacent (C_β_)–H atoms resulted in a nonplanar wavy PDSSTV conformation, UV–vis absorption spectroscopy revealed the presence of strong intermolecular interactions between polymer chains. These interactions were believed to arise from strong Se–Se interactions caused by the large size and polarizability of Se orbitals in DSS. In addition, annealing of the polymer film at 250 °C resulted in a marked crystallinity increase and promoted highly ordered edge‐on orientation and thus facilitated charge transport in the corresponding OFETs. The use of a PS/P(VDF‐TrFE‐CFE) dielectric system in OFETs allowed one to achieve a maximum saturation mobility of 2.1 cm^2^ V^−1^ s^−1^ at a voltage (−30 V) much lower than those required for most other high‐mobility OFETs. Therefore, it was concluded that the utilization of large chalcogen‐containing fused aromatic systems promotes the formation of strong interchain interactions and thus holds great promise as a method of obtaining high‐mobility conjugated polymers.

## Conflict of Interest

The authors declare no conflict of interest.

## Supporting information

Supplementary
