# Supplementary material for: Diseleno[3,2‐b:2′,3′‐d]selenophene‐Containing High‐Mobility Conjugated Polymer for Organic Field‐Effect Transistors
Source: Adv Sci (Weinh). 2019 Apr 26;6(13):1900245. doi: 10.1002/advs.201900245 (PMC6662335; doi:10.1002/advs.201900245)
Supplement: Supplementary file 1 — Supplementary [file ADVS-6-1900245-s001.pdf]

Copyright WILEY-VCH Verlag GmbH & Co. KGaA, 69469 Weinheim, Germany, 2019.

ADVANCED SCIENCE
Open Access

Supporting Information

for *Adv. Sci.,* DOI: 10.1002/advs.201900245

Diseleno[3,2-*b*:2',3'-*d*]selenophene-Containing High-Mobility Conjugated Polymer for Organic Field-Effect Transistors

Soo-Young Jang, In-Bok Kim, Minji Kang, Zuping Fei, Eunhwan Jung, Thomas McCarthy-Ward, Jessica Shaw, Dae-Hee Lim, Yeon-Ju Kim, Sanjay Mathur, Martin Heeney, and Dong-Yu Kim**

WILEY-VCH

Copyright WILEY-VCH Verlag GmbH & Co. KGaA, 69469 Weinheim, Germany, 2019.

Supporting Information

Diseleno[3,2-*b*:2',3'-*d*]selenophene–Containing High-Mobility Conjugated Polymer for Organic Field-Effect Transistors

Soo-Young Jang, In-Bok Kim, Minji Kang, Zuping Fei, Eunhwan Jung, Thomas McCarthy-Ward, Jessica Shaw, Dae-Hee Lim, Yeon-Ju Kim, Sanjay Mathur, Martin Heeney, and Dong-Yu Kim**

Contents

S1. Materials and general procedures
S2. Experimental section
S3. NMR spectra of monomers and PDSSTV
S4. Gel permeation chromatography (GPC) analysis of PDSSTV
S5. Thermal properties of PDSSTV
S6. Cyclic voltammetry of PDSSTV
S7. Calculated molecular orbitals and energy levels in density functional theory
S8. Gate voltage independence of PDSSTV in OFETs
S9. OFET devie performance of dithieno[3,2-*b*:2',3'-*d*]thiophene-based polymer
S10. OFET devie performance at bottom gate/bottom contact (BG/BC) configuration
References

S1. Materials and general procedures

All chemical reagents, P(VDF-TrFE-CFE) and PS were purchased and used as received. Tetrahydrofuran (THF) as the reaction solvent was distilled from a sodium/benzophenone system prior to use. Di-trimethyl-stannyl functionalized TV (**6**)[1] was synthesized via published literature procedures. The ^{1}H NMR spectrum was recorded on a Bruker AV-400 (400 MHz), using $CDCl_3$. Chemical shifts were reported as parts per million relative to an internal TMS standard. Elemental analysis was recorded on an EA1112 instrument (CE Instrument). The number-average molecular weight (M_n) and weight-average molecular weight (M_w) were determined using high temperature gel permeation chromatography (Polymer Laboratories PL-GPC 210) with 1,2,4-trichlorobenzene as the eluent (flow rate of 1 mL/min, at 150 °C) and were calibrated using a narrow polydispersity polystyrene as the standard. Thermal gravity analyses (TGA) were carried out on a TA Instrument TGA-Q50 instrument at a heating rate of 10 °C/min in a nitrogen atmosphere, and differential scanning calorimetry (DSC) analyses were performed on a TA Instruments DSC-Q20 instrument at a heating rate of 10 °C/min in a nitrogen atmosphere. Absorption spectra were recorded on a Varian Cary 5000 UV-Vis-NIR Spectrophotometer. Cyclic voltammetry measurements of the polymers were performed using an AUTOLAB Eco Chemie instrument at a scan rate of 50 mV/s. The samples of polymers were measured using indium tin oxide (ITO) as the working electrode, a Pt wire as the counter electrode, and a Ag/AgCl as the reference electrode in an acetonitrile solution with 0.1 M tetrabutylammonium hexfluorophosphate as the supporting electrolyte (Ferrocene -4.8 eV). GIXD was measured using the synchrotron radiation sources 9A beam line at the Pohang Accelerator Laboratory (PAL). OFET device fabrication and characterization were performed under a nitrogen atmosphere.

S2. Experimental section

S2.1. Synthesis of 2,6-dibromodiselenopheno[3,2-*b*:2',3'-*d*]selenophene (**5**)

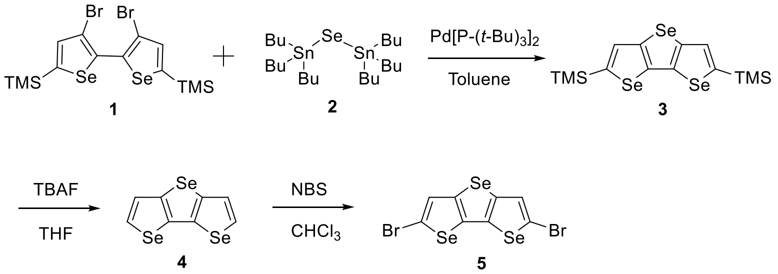

Scheme **S1**. Synthetic schemes for **5**

1) 1,1,1,3,3,3-hexabutyldistannaselenane (**2**)

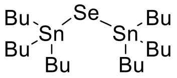

A 500 mL round-bottomed flask was charged with a stirrer bar, sodium (1.21 g, 52.4 mmol), selenium powder (2.07 g, 26.2 mmol), naphthalene (0.6 g, 4.7 mmol), and dry THF (250 mL). The mixture was heated to reflux for 15 h, resulting in the formation of a white suspension. The solution was cooled to 0 °C and tributylchlorostannane (17.1 g, 52.4 mmol) was added in one portion. The mixture was allowed to warm to RT and stirred for 12 h. Toluene (100 mL) was added and the mixture was filtered to remove the sodium chloride. The solvents were removed under reduced pressure and the resulting oil was warmed under vacuum (c.a. 10 mbar) to remove the naphthalene through sublimation. The resultant oil was found to be 77% product and 23% unreacted tributylchlorostannane (by GC/MS). The tributylchlorostannane was removed by passing through a column consisting of c.a. 25% by volume potassium fluoride in silica to give the target compound as a very pale yellow oil (9.972 g, 15.1 mmol, 57.7%). ^{1}H NMR ($CDCl_3$, 400 MHz): ∂ (ppm) 1.52 (dt, *J*=15.8, 7.4 Hz, 2H), 1.43-1.26 (m, 2H), 1.12 (t, *J*=8.2 Hz, 2H), 0.91 (t, *J*=7.4 Hz, 3H); ^{13}C-{^{1}H} NMR ($CDCl_3$, 100 MHz): ∂ (ppm) 29.1, 27.2, 15.6; MS (HR EI): *m/z* calculated for $C_{24}H_{54}SeSn_2$: 662.1435 (M^+); found 662.1447.

2) 2,6-bis(trimethylsilyl)diselenopheno[3,2-*b*:2',3'-*d*]selenophene (DSS-TMS) (**3**)

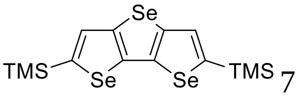

A 10-20 mL microwave vial was charged with a stirrer bar, 5,5'-bis(trimethylsilyl)-3,3'-dibromo-2,2'-biselenophene[1] (**1**, 1.00 g, 1.78 mmol), 1,1,1,3,3,3-hexabutyldistannaselenane (**2**, 1.17 g, 1.78 mmol), bis(tri-*t*-butylphosphine) palladium(0) (45 mg, 0.09 mmol), and dry toluene (10 mL); the tube was then sealed with a septum-seal. Argon was bubbled through the stirred mixture at RT for 0.5 h. The mixture was then heated to 135 °C for 12 h with constant stirring after which it was allowed to cool to RT and poured into distilled water (50 mL). The mixture was extracted with hexanes (3×20 mL), and the combined organic extracts were washed with distilled water (3×20 mL) and brine (50 mL) and dried over anhydrous $MgSO_4$. The crude product was purified by column chromatography on silica eluting with hexanes : ethyl acetate (1:0 – 97:3) followed by recrystallization from ethanol to give the target compound as colourless needles (0.41 g, 0.85 mmol, 48%). ^{1}H NMR ($CDCl_3$, 400 MHz): ∂ (ppm) 7.66 (s, 2H), 0.36 (s, 18H); ^{77}C-{^{1}H} NMR ($CDCl_3$, 100 MHz): ∂ (ppm) 149.1, 142.8, 139.8, 132.0, 0.2; MS (HR EI): *m/z* calculated for $C_{14}H_{20}Si_2Se_3$: 483.8599 (M^+); found 483.8610.

3) diselenopheno[3,2-*b*:2',3'-*d*]selenophene (DSS) (**4**)

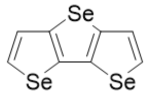

DSS-TMS (**3**, 0.38 g, 0.79 mmol) was dissolved in 30 mL of dry THF. The mixture was cooled to 0 °C whereupon tetrabutylammonium fluoride solution (1 M in THF, 1.75 mL, 1.75 mmol) was added in one portion with stirring. The mixture was stirred at 0 °C for 10 min before being raised to RT and stirred for a further 40 min. The reaction mixture was poured into saturated ammonium chloride solution (100 mL) and then extracted with diethyl ether (3×25 mL). The combined organic extracts were washed with distilled water (5×20 mL) and brine (50 mL) and then dried over anhydrous $MgSO_4$.The solvents were removed under reduced pressure and the crude product purified by flash column chromatography on silica (eluent: hexane) to give the target compound as large colourless crystals (0.19 g, 0.56 mmol, 71%). ^{1}H NMR ($CDCl_3$, 400 MHz): ∂ (ppm) 7.94 (d, *J*=5.6 Hz, 2H), 7.56 (d, *J*=5.6 Hz, 2H); ^{13}C-{^{1}H} NMR ($CDCl_3$, 100 Mhz): ∂ (ppm) 140.5, 135.4, 129.4, 125.9; MS (HR EI): *m/z* calculated for $C_8H_4Se_3$: 339.7809 (M^+); found 339.7815.

4) 2,6-dibromodiselenopheno[3,2-*b*:2',3'-*d*]selenophene (**5**)

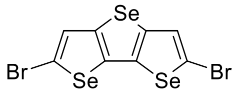

DSS (**4**, 0.45 g, 1.33 mmol) was dissolved in 50 mL of dry chloroform at RT. NBS (0.48 g, 2.66 mmol) was added in one portion under dark. The mixture was stirred for overnight. Saturated Na_2SO_3 solution (20 mL) was added and then extracted with chloroform (3×30 mL). The combined organic extracts were washed with distilled water (5×40 mL) and then dried over anhydrous $MgSO_4$.The solvents were removed under reduced pressure. The crude product was purified by recrystallization from DCM to afford the target compound as off-white needle crystals (0.52 g, 1.05 mmol, 79%). ^{1}H NMR ($CDCl_3$, 400 MHz): ∂ (ppm) 7.50 (s, 2H); ^{13}C-{^{1}H} NMR ($CDCl_3$, 100 Mhz): ∂ (ppm) 138.3, 136.0, 128.6, 113.9; MS (HR EI): *m/z* calculated for $C_8H_2Br_2Se_3$: 495.6019 (M^+); found 495.6001. Elemental Anal. Calcd (%) : C, 19.42; H, 0.41. Found (%) : C, 19.49; H, 0.43.

S2.2. Synthesis of 5,5’-ditrimethylstannyl-(E)-1,2-(3,3′-dioctadecyl-2,2′-dithienyl)ethylene (**6**)[2]

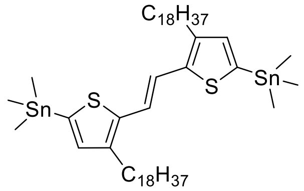

^{1}H NMR ($CDCl_3$, 400 MHz): ∂ (ppm) 6.99 (s, 2H), 6.90 (s, 2H), 2.64 (t, 4H), 1.24–1.4 (m, 64H), 0.87 (t, 6H), 0.28–0.42 (m, 18H). ^{13}C NMR (CDCl3, 100 MHz, ppm): 142.08, 141.69, 138.14, 135.25, 119.41, 31.92, 31.12, 29.70, 29.66, 29.63, 29.54, 29.50, 29.36, 28.36, 22.69, 14.12, -8.28. Elemental Anal. Calcd (%) : C, 61.06; H, 9.46; S, 6.27. Found (%) : C, 61.16; H, 9.51; S, 6.36.

S2.3. Synthesis of PDSSTV

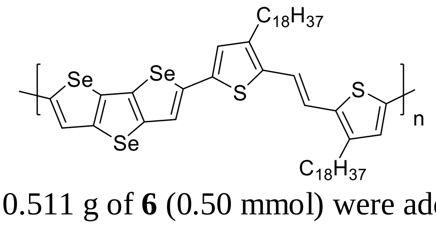

0.247 g of **5** (0.50 mmol) and 0.511 g of **6** (0.50 mmol) were added to ~5 mL microwave vessel (Biotage) with a magnetic stir bar and dissolved in 8 ml anhydrous chlorobenzene.

$Pd_2(dba)_3$ (9.16 mg, 2 mol %) and $P(o\text{-}tol)_3$ (12.2 mg, 8 mol %) were added to the vial and sealed. The vessel was vac-N_2 purged for three times and placed in the microwave reactor. The sealed vessel was heated step-wise as 100, 120, 140, 160 ^oC for 2, 2, 2, 40 min. After cooling to room temperature, the polymer solution was precipitated into the aq. HCl/methanol (5 v%) solution and stirred for 2 h. After filtered with soxhlet timble, the polymer was purified with methanol, hexane via Soxhlet extraction with a final extraction in chloroform. The solution was precipitated into 200 ml of methanol and filtered to afford 0.409 g (yield: 79.1 %). M_n 19.1 kDa, M_w/M_n 2.0, Elemental Anal. calcd: for $(C46H64NS5)_n$: C, 63.44; H, 8.18; S, 6.05; found: C, 63.35; H, 8.15; S, 6.03. ^1H NMR (C_6D_5Cl, 500 MHz): ∂ (ppm) 7.30 (br, 2H), 6.84 (br, 2H), 6.79 (br, 2H), 2.6 (br, 2H), 1.2–1.8 (br, 64H), 0.88 ppm (6H, br).

S2.4. Fabrication and characterization of the OFETs

Top-gate/bottom-contact OFETs were fabricated. Au/Ni (14/4 nm) (Ni was the adhesion layer) were patterned as the source and drain electrodes [channel width (W): 1 mm, channel length (L): 20 μm] by conventional photolithography on Corning Eagle 2000 glass substrates. After sequentially cleaning the patterned substrates using ultra-sonication in DI, acetone and isopropyl alcohol, the residual solvents were dried in a conventional oven. Before coating the polymer solution, the substrates were treated with UV ozone exposure for 20 min. PDSSTV solution was produced using 1,2-dichlorobenzene as a solvent yielding 7 mg/ml solution and was spin-coated onto a substrate to form a thickness of ~ 30 nm under a nitrogen atmosphere. Some of the polymer films were thermally annealed at 250 °C for 20 min and some were not. Polystyrene was used for the 1st gate dielectric layer at a concentration of 2.5 mg/ml in 2-butanone. The solution was spin-coated with 2000 rpm for 60 s (accelerated for 3.0 s) and the film was thermal annealed at 80 ^oC for 2 h. Next, the 2nd gate dielectric layer, P(VDF-TrFE-CFE), was deposited under the concentration of 30 mg/ml in acetonitrile. The solution was spin-coated with 2000 rpm for 60 s (accelerated for 3.0 s) and the film was thermal annealed at 80 ^oC for 2 h. The capacitance of bi-layered dielectrics was 48.9 nF/cm^2. Finally, the OFETs were completed by thermal evaporation of the Al gate electrode (50 nm) under high vacuum condition (1.0 x 10^{-6} torr) using a metal shadow mask. The electrical characteristics of the fabricated OFET devices were measured using a Keithley 4200-SCS instrument in a glove-box under a nitrogen atmosphere. The μ_{FET} and V_{Th} were calculated at the saturation region using a gradual-channel approximation equation.

S3. NMR spectra of monomers and PDSSTV

2,6-dibromodiselenopheno[3,2-*b*:2',3'-*d*]selenophene (**5**)

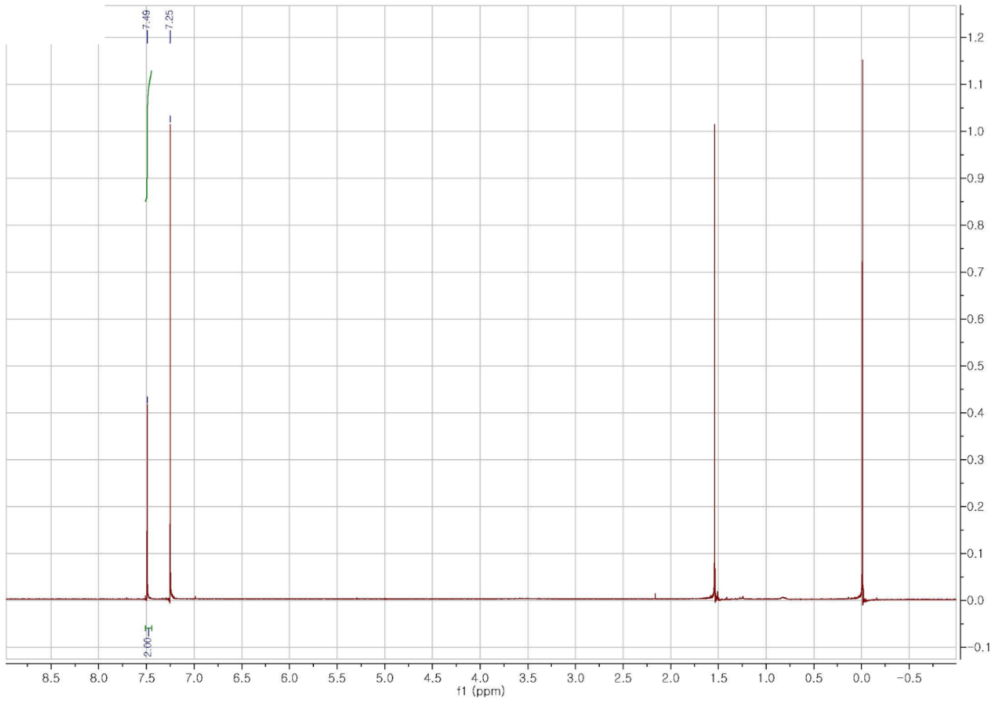

Figure. S1 ^{1}H-NMR spectrum of 2,6-dibromodiselenopheno[3,2-*b*:2',3'-*d*]selenophene (**5**) in $CDCl_3$.

5,5′-Ditrimethylstannyl-(*E*)-1,2-(3,3′-dioctadecyl-2,2′-dithienyl)ethylene (**6**)

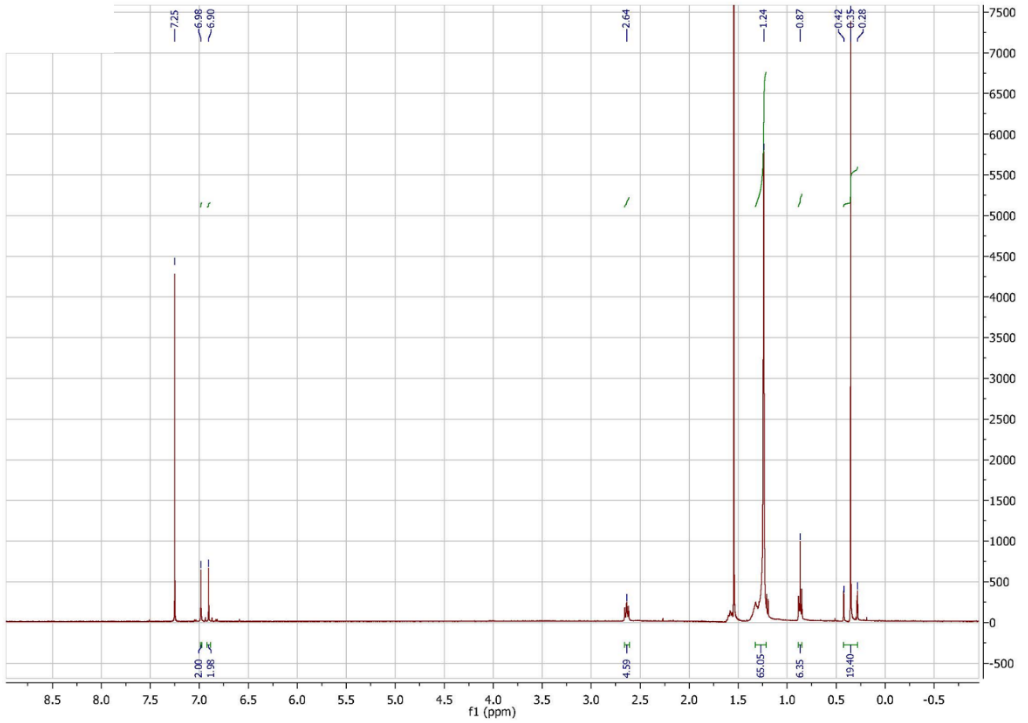

Figure. S2 ^{1}H-NMR spectrum of 5,5’-ditrimethylstannyl-(*E*)-1,2-(3,3′-dioctadecyl-2,2′-dithienyl)ethylene (**6**) in $CDCl_3$.

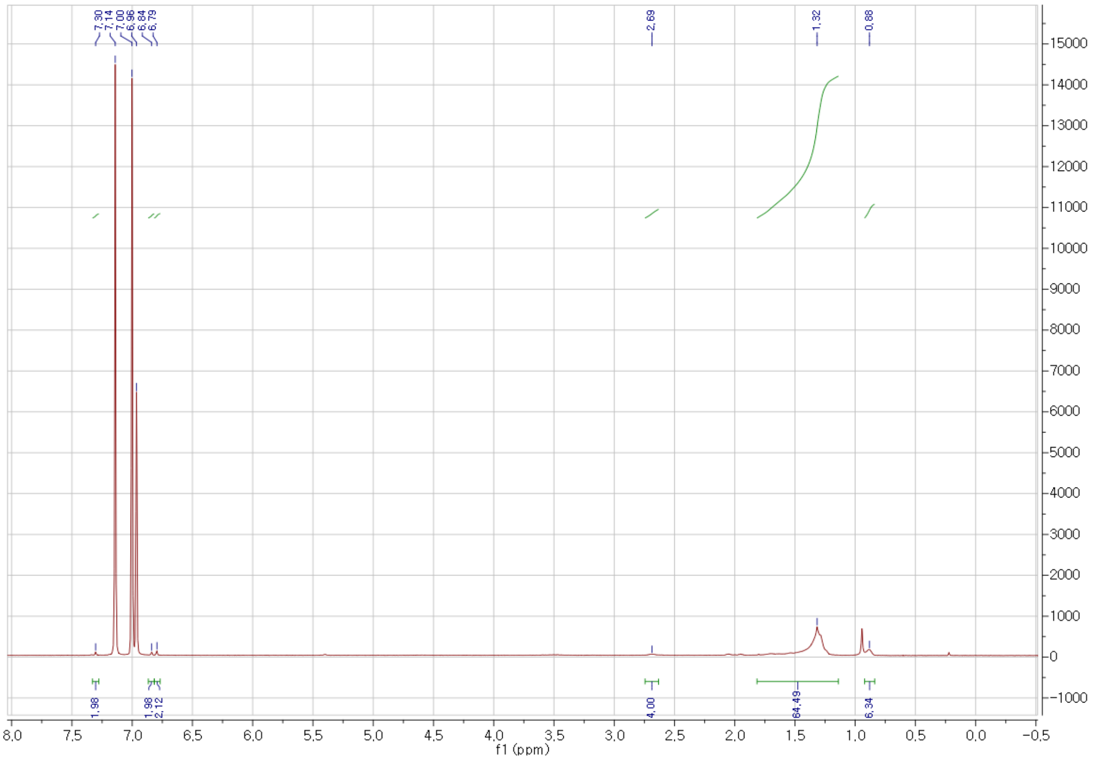

Figure. S3 ^{1}H-NMR spectrum of PDSSTV in C_6D_5Cl at 80 °C.

S4. Gel permeation chromatography (GPC) analysis of PDSSTV

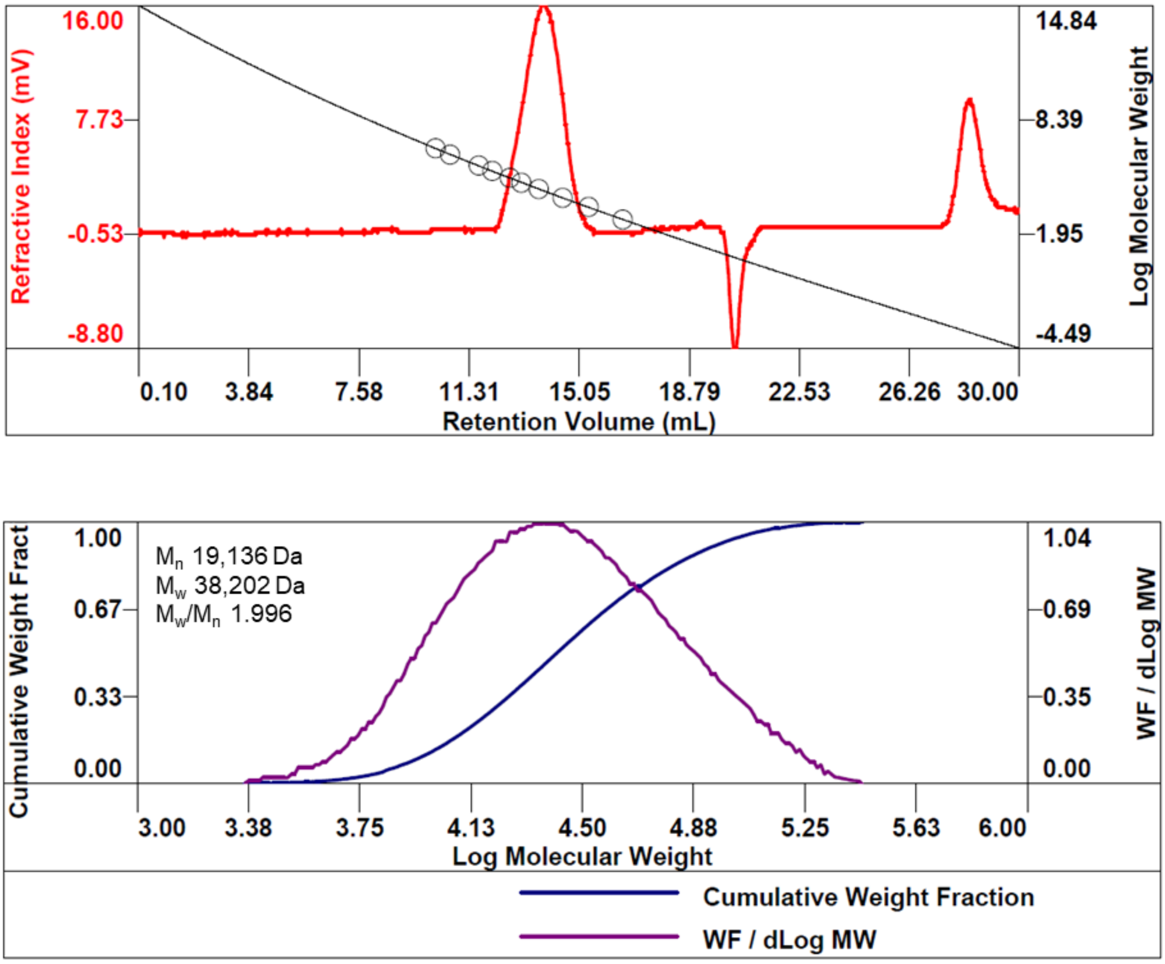

Figure. S4 Gel permeation chromatography analysis result of PDSSTV.

S5. Thermal properties of PDSSTV

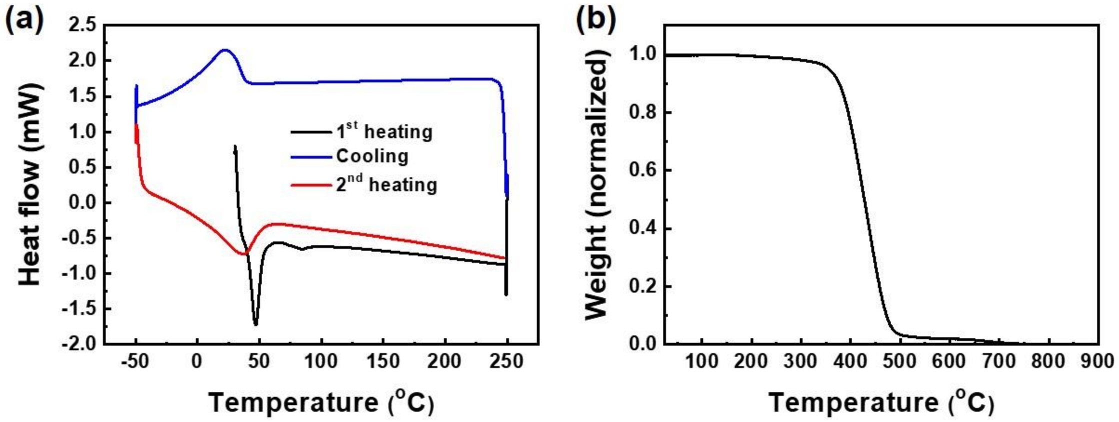

Figure. S5 Thermograms of differential scanning calorimetry (a) and thermal gravity analysis (b) of PDSSTV.

S6. Cyclic voltammetry of PDSSTV

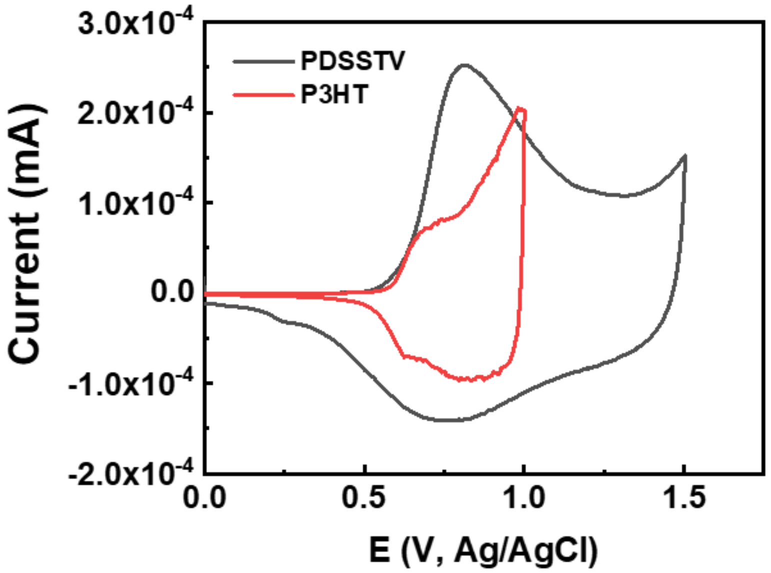

Figure. S6 Cyclic voltammogram of PDSSTV (with P3HT as a reference material).

S7. Calculated molecular orbitals and energy levels in density functional theory

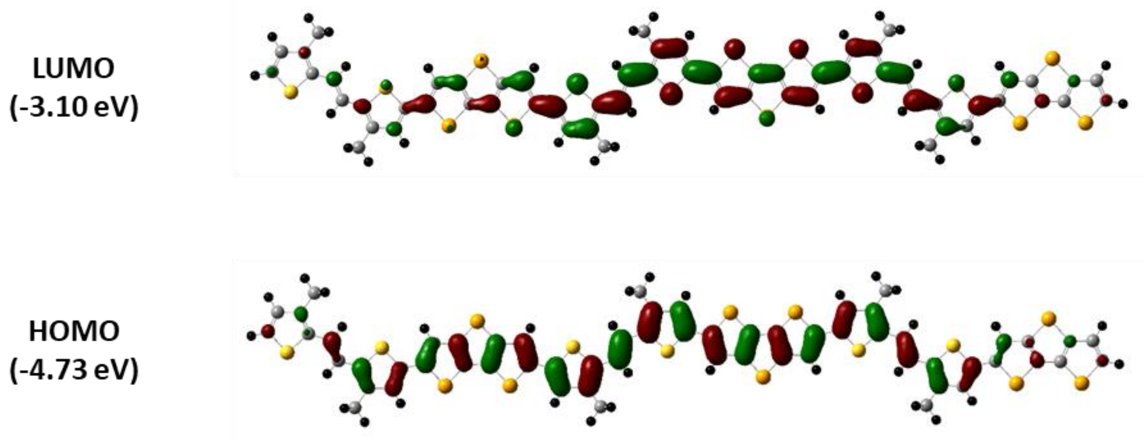

Figure. S7 Calculated HOMO and LUMO of the trimer (Simulation model: B3LYP-D3/6-311++G*).

S8. Gate voltage independence of PDSSTV in OFETs

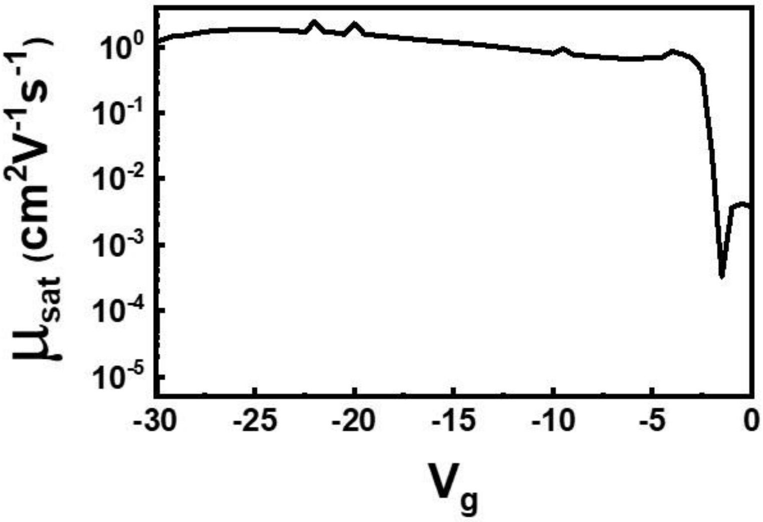

Figure. S8 Saturated mobility *vs.* Gate voltage of PDSSTV-based OFETs.

S9. OFET devie performance of dithieno[3,2-*b*:2',3'-*d*]thiophene-based polymer

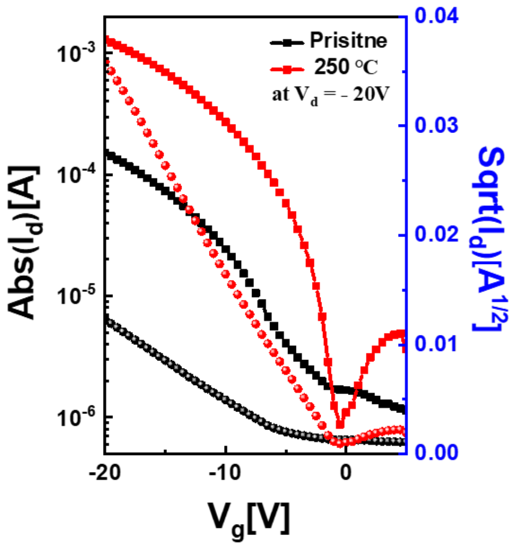

Figure. S9 Transfer curves of OFETs using dithieno[3,2-b:2',3'-d]thiophene-based polymer (pristine, 250 °C).

Table S1. Summary of OFETs performance using dithieno[3,2-b:2',3'-d]thiophene-based polymer

Annealing temperature [°C]	Average mobility [cm^2 $V^{-1}s^{-1}$]	Standard deviation	Threshold voltage [V]
pristine	0.44	0.13	-3.88
250	2.03	0.40	0.16

* -20 V_g is the voltage limit of this device. (Above -20 V_g, the devices failed.)
* Channel length/Width: 20 µm / 1.0 mm

S10. OFET devie performance at bottom gate/bottom contact (BG/BC) configuration

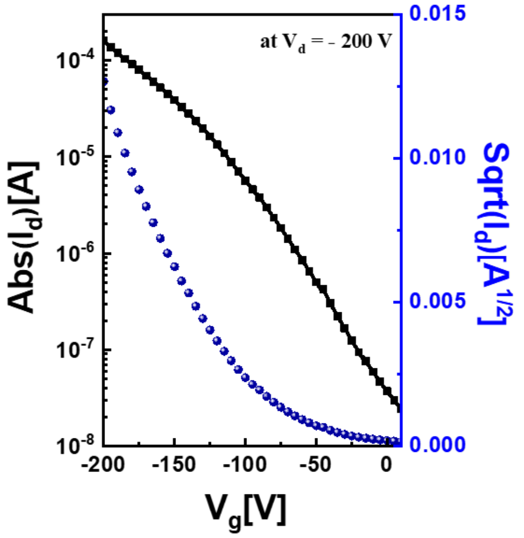

Figure. S10 Transfer curve of PDSSTV-based BG/BC OFETs (250 °C).

Table S2. Summary of BG/BC OFETs performance

Annealing Temperature [°C]	Mobility [$cm^2\ V^{-1}s^{-1}$]	Threshold voltage [V]
250	0.12	-132

* Devices were fabricated on Hexamethyldisilazane (HMDS) modified 300 nm SiO_2 wafers.
* Channel length/Width: 20 μm / 1.0 mm

References

1. Z. Fei, Y. Han, E. Gann, T. Hodsden, A. S. R. Chesman, C. R. McNeill, T. D. Anthopoulos and M. Heeney, *J. Am. Chem. Soc.* 2017, **139**, 8552−8561.
2. S.-Y. Jang, I.-B. Kim, J. Kim, D. Khim, E. Jung, B. Kang, B. Lim, Y.-A. Kim, Y. H. Jang, K. Cho and D.-Y. Kim, *Chem. Mater.*, 2014, **26**, 6907-6910.
